# Application of the Recognition Algorithm of News Sentiment Dissemination Tendency Based on Multi-Mode Information Fusion

**DOI:** 10.3389/fpsyg.2022.853899

**Published:** 2022-05-31

**Authors:** Makiko Chiba

**Affiliations:** College of Foreign Languages, Zhejiang University of Technology, Hangzhou, China

**Keywords:** multi-modal, information fusion technology, news sentiment communication, tendency recognition algorithm, news sentiment dissemination

## Abstract

Since the popularity of the Internet, it is now customary for people to browse the news on the Internet. In the process of browsing news, there are often comments to express and spread personal emotions. This has become a very popular way of spreading news sentiment. This research is based on information fusion technology to realize the research on the identification algorithm of news sentiment communication tendency. Based on the research results of many scholars, this article first elaborates the definition, method, and framework of information fusion technology. In this paper, the corresponding technology is selected, and then in the research of the tendency recognition algorithm, an emotion tendency recognition algorithm based on the integrated probabilistic reasoning model is proposed. The algorithm proved its effectiveness and robustness through experiments. Finally, the experimental results show that in all the parameter combination conditions, the algorithm can not only reduce the feature dimension but also ensure the accuracy of recognition under the probability of 89%.

## Introduction

As information explodes in the new era, it has become an indispensable technology for information convergence in all fields. Simultaneously, as people pay more and more attention to online news, which has led to an increasing informativeness of news commentary. And people’s comments are often mixed with many personal emotions, so the recognition of news sentiment tendencies has become an inevitable result. The work of this article can not only analyze the changing trend of emotional orientation, but also provide an indispensable reference for the future news industry.

Many scholars at home and abroad have provided a large number of references for the research on multi-mode, information fusion, news sentiment communication, and tendency recognition algorithms. Peng et al. (2017) proposed a semi-supervised learning method based on the collaborative filtering theory using labeled and unlabeled interactive information, called NormMulInf. The method he proposed initially determined the similarity measure by integrating biological information. For example, the similarity between samples and the local correlation between sample labels. He then integrated the similarity information into a robust principal component analysis model, which was solved using augmented Lagrangian multipliers. Experimental results show that the method he proposed can accurately classify and predict drug–target interactions. Hong et al. (2017) proposed a new framework called Multi-scale Spatial Information Fusion (MSIF). The MSIF he proposed captures the inherent spatial information contained in homogeneous regions of different sizes through a multi-scale strategy. In MSIF, local one-dimensional embedding (L1-DE) and information fusion are used iteratively, and the iterative process is terminated within a finite number of steps. Algorithm analysis proves the effectiveness of the proposed method. Experimental results on four widely used HSI data sets show that compared with other state-of-the-art spectral spatial classification methods, his proposed method achieves higher classification accuracy. Li et al. (2018) proposed a Bayesian information fusion method for determining the probabilistic health index of power transformers. The method he proposed integrates various data obtained from transformer measurements, maintenance records, and fault statistics. By using these data, he used Bayesian Belief Network (BBN) to build an inference model. In the inference model, the significance of each individual measurement to the corresponding component in the BBN is jointly determined by principal component analysis and expert experience, and then quantified by score probability transformation. Finally, the reasoning model is used to derive the probabilistic health index, and he provides a case study to prove the applicability of the proposed method for assessing the condition of the transformer. Qi et al. (2017) proposed a laser beam welding (LBW) process parameter optimization method based on multi-fidelity (MF) meta-model. The results show that the MF meta-model is superior to the single-fidelity meta-model in terms of global and local accuracy. Finally, the fast elite non-dominated sorting genetic algorithm (NSGA-II) is used to facilitate the exploration of the parameter space of the LBW process and the multi-objective pareto optimal search. Lei et al. (2021) proposed a novel graph convolution model to learn and fuse multi-hop neighbor information relations. Lei et al. (2021) uses a weight sharing mechanism to design graph convolutions of different orders to avoid potential overfitting problems. In addition, Lei et al. (2021) designed a new multi-hop neighbor information fusion (MIF) operator that mixes different neighbor characteristics from 1 hop to k hops. Lei et al. (2021) theoretically analyzed the computational complexity of the model and the number of trainable parameters. Experiments on the text network show that his proposed model achieves the most advanced performance than text GCN. Ouyang et al. (2021) proposed an information fusion FMEA method based on binary language information and interval probability. He uses the theory of binary language sets to convert heterogeneous information into interval numbers, and at the same time uses interval probability comparison to analyze failure modes. Finally, he verified the reliability and effectiveness of the proposed method by comparing different FMEA methods. The data of these studies are not comprehensive, and the experimental results are still open for discussion and are not recognized by the public, so they cannot be popularized.

This paper’s innovation was in reducing the data storage space by PCA dimensionality decrease, which resulted in accelerating the algorithm. While this paper employs an approach, model, and architecture for data fusion technique, which are most appropriate for this study are chosen. This provides the most effective technical support for research. Finally, this paper proposes an emotional orientation recognition algorithm based on an integrated probabilistic inference model, which significantly improves the efficiency of emotional orientation recognition.

## Information Fusion and the Tendency of News Sentiment Communication

### Information Fusion

Analysis and processing over a large amount of accessed information is the process of information fusion to achieve the target result. From the perspective of mathematics, information fusion is to find the relationship between multiple functions and solve them in parallel to obtain the output (Huang, 2017).

The functional model of multi-modal information fusion includes a variety of functional modules (Prasath, 2017). According to the level of information in the transmission process, it is divided into low-level processing and high-level processing. In the early stage of the low-level processing process, a numerical result is formed by mining the characteristics of the information data. It is possible for these results to reflect the underlying signal signatures in multi-modal information which enables correlation and identification between data. That data can be used as a basis for the analysis of information. Additionally, since the advanced processing is also analyzed at a later stage of information fusion, on a semantic level, it reflects the features of the semantic level. It mainly extracts information features from symbolic logic and can realize functional modules, such as logical reasoning and situation estimation (Chahine et al., 2018; Yu et al., 2019).

The signal information processing process is information data processing, feature extraction, state recognition, and decision-making judgment (Groen et al., 2017). Regarding the information involved in each stage of the information processing process, there are three different stages of information processing. This first stage is data, which is processed by smoothing and filtering the collected data; at the second stage is features, which extract the characteristics of the information; from the third stage is decision-making. The three levels of information fusion have their own characteristics and will not conflict with each other in the process. It can perform fusion processing on information at any level, but it will increase the load of the system while increasing the depth of information fusion processing. Therefore, it should be judged according to the task’s information fusion requirements, hardware processing capabilities, etc., and decide at which level the fusion processing should be performed (Chandra et al., 2019).

In the first layer of fusion, data layer fusion is the first layer of fusion. It performs data fusion immediately after collecting the original data. The characteristic is less amount with information loss and better accuracy of fusion results. However, due to the complexity and noise of the collected data, the amount of fusion calculation is large, and the requirements for information synchronization are relatively high; the feature layer is merged into the second level. After data processing, the information expressed by the data has become more pure. Therefore, feature extraction can be performed on the information, and the obtained feature vectors can be processed uniformly for judgment. The calculation amount of this fusion method is relatively small, but the fusion result is greatly affected by the characteristic information, and it is susceptible to interference; the fusion processing time of the decision-making layer is the preliminary detection result that the system has formed on the target. At this time, the decision-level fusion processing can allow the system to merge the single-source detection results to form a more complete and accurate judgment. This method has the least amount of calculation and the strongest anti-interference ability. However, due to problems, such as the difficulty of obtaining prior knowledge and the difficulty of constructing huge knowledge bases, related theories need to be further improved (Song et al., 2017).

Commonly used multi-mode information fusion methods can be roughly divided into three categories: probability and statistics methods, logical reasoning methods, and neural network methods (Gupta and Banerjee, 2019). A probabilistic and statistical approach to the problem includes the weighted average method, the Bayesian estimation method, or the Kalman filtering method. There are specific mathematical models for all of them, which are traced for the process from fusion information manipulation. But they are not good at handling massive data, and the difficulty of solving will increase with the increase of the amount of data, and it is necessary to adopt coefficients suitable for the system according to the requirements of different systems or prior knowledge. When the model does not conform, the fusion result will be far from the actual value. Logical reasoning methods are usually D-S evidence theory and fuzzy logic methods, which are closer to human thinking than probability and statistics methods. It is usually used for information fusion at the decision-making level, but evidence theory requires evidence to be independent of each other. However, in complex systems, there are many coupling relationships between various evidence, and there may be exponential explosion problems under the premise of large amount of data. The fuzzy logic method needs to determine the fuzzy language set and the membership function corresponding to the actual input distribution space, and the adaptive ability is too poor. The neural network method has strong fault tolerance and self-learning ability. Through its powerful generalization ability and non-linear processing ability, it can theoretically fit arbitrarily complex functions. It obtains the hidden knowledge in the information through different learning algorithms, and obtains the uncertainty reasoning mechanism. However, the neural network method also needs to rely on human prior knowledge to adjust the network architecture. Each of these multi-mode information fusion methods has its own characteristics and shortcomings. Due to the complementarity of various methods, each method is often combined to construct a new information fusion method (Kelly and Ahmad, 2018).

Commonly used models for information fusion include JDL model, Dasarathy model, waterfall model, etc. This article briefly introduces two representative models of JDL and Dasarathy (Minchae and Kyung-Shik, 2017; Boguslaw et al., 2018).

#### JDL Model

**Figure 1:**
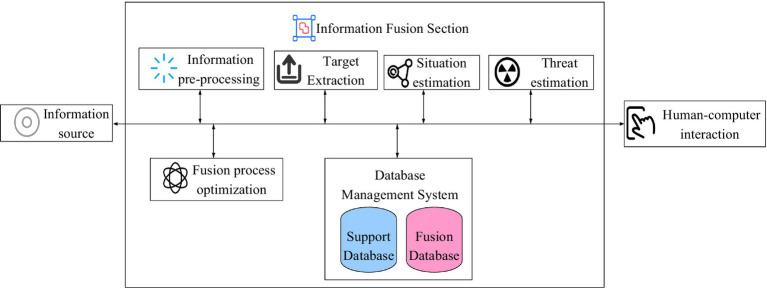
JDL data fusion model.

The JDL model is a fusion model constructed as a clue to the information processing process. With a general sense, the JDL fusion model is a functional model by which the basic concepts and processes of information fusion are explained and provides that framework for further discussion. Although the original intention of this model is based on the needs of military applications, it has been developed and perfected and is also used in civil applications (Yadav et al., 2019). The JDL model is shown in Figure 1.

#### Dasarathy Model

**Figure 2:**
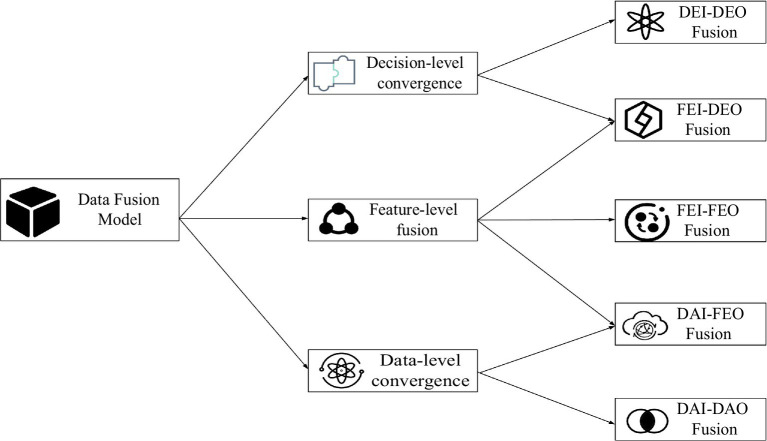
Dasarathy data fusion model.

With the Dasarathy model, it is built based on the perspective of information representation. It can demonstrate clearly and effectively the levels of fusion behavior (Yang and Seng, 2017). The Dasarathy model is shown in Figure 2.

**Figure 3:**
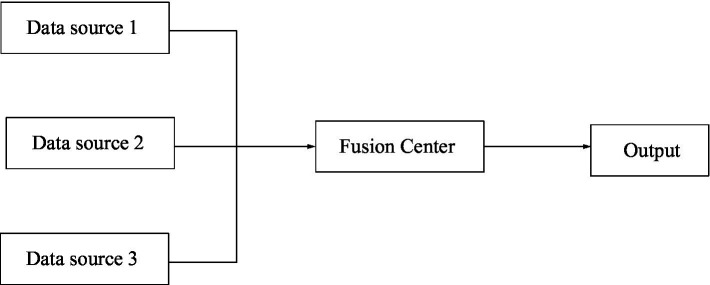
Centralized structure of information convergence.

The information fusion system architecture has not yet reached a unified classification form. But as a whole, it can be divided into three categories: centralized structure, distributed structure, and hierarchical structure (Allen et al., 2019). The so-called centralized structure means that all fusion processes are completed by a fusion center. The centralized system structure is shown in Figure 3.

**Figure 4:**
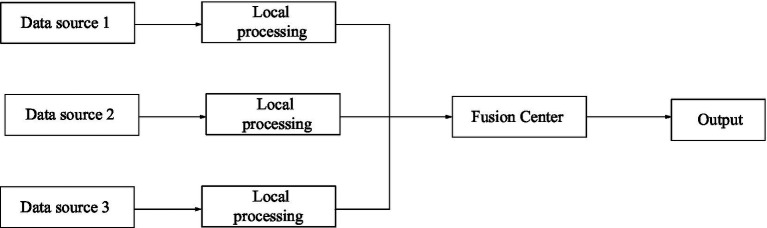
Distributed architecture of information fusion.

Compared with the centralized structure, the distributed structure requires almost no original information and data, so the requirements for channel capacity are greatly reduced (Chen and Chen, 2019). Its structure is shown in Figure 4.

**Figure 5:**
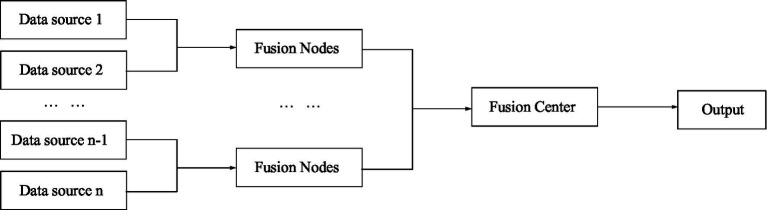
Information fusion without feedback hierarchical structure.

**Figure 6:**
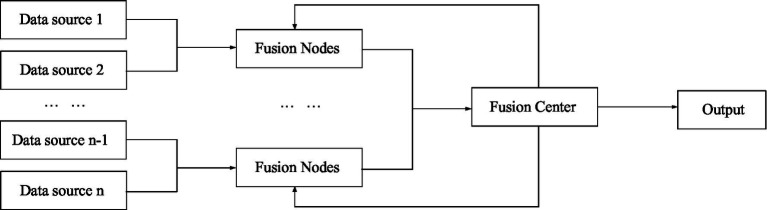
Information fusion with feedback hierarchical structure.

Hierarchical structure is a combination of centralized structure and distributed structure. The hierarchical structure is divided into two types, one is without feedback and the other is with feedback (Yang et al., 2017; Kamel et al., 2019). Figure 5 shows a hierarchical structure without feedback, and Figure 6 shows a hierarchical structure with feedback.

### Emotional Orientation Recognition Algorithm Based on Integrated Probabilistic Reasoning Model

#### PCA Dimensionality Reduction

Proportional component analysis (PCA) was a popular dimensionality reduction algorithm that was widely being used in the field of image recognition (e.g., face recognition). That kind of algorithm has been developed to solve the problem of temporal complexity and spatial complexities which caused by the high-level dimensionality in the feature space. The principle of PCA is to linearly transform the high-dimensional feature space to generate a low-dimensional feature space to achieve the purpose of reducing the feature dimension. For example, if there is “I” in each sample, or almost all texts have “I,” then “I” is not very distinguishable. The contribution to classification is small and can be removed. Therefore, the purpose of PCA dimensionality reduction is to find those elements with large changes, that is, those dimensions with large variance. The removal of those dimensions that have not changed much reduces the amount of calculation and improves the efficiency and accuracy of classification (Mostafa and Nebot, 2017).

The linear transformation of PCA is not arbitrary. The low-dimensional feature space obtained after linear transformation must be able to represent the main information of the original high-dimensional feature space, so that the transformation is not distorted. So during dimensionality reduction in PCA, noise and redundant features are removed such that the correlation across dimensions in the generated low-dimensional feature space to be as small as possible. Each dimension is highly representative and contains richer classification and identification information. After dimensionality reduction, the dimensional correlation in the feature space should be as small as possible, and the covariance matrix between the dimensions of the original feature space needs to be calculated in the linear transformation. The elements in the covariance matrix are composed of the covariance between each vector element. After obtaining the covariance matrix, the eigenvalues and eigenvectors are further calculated to obtain the principal components.

#### Emotional Orientation Recognition Algorithm Based on Integrated Probabilistic Reasoning Model

##### Dictionary-Based Emotion Recognition Algorithm

The research on the recognition method of review text sentiment orientation mainly starts from the three levels of vocabulary, sentence and text. In the study of vocabulary-level emotional orientation recognition, the recognition method based on dictionary is an important research point. An algorithm for dictionary-based sentiment recognition is constructed in this paper using a simple “numerical” approach. They are namely to determine the emotional orientation of a review text with a comparison of the number of its positive and negative emotional comments.

###### Dictionary Selection

This article reviews the text sentiment orientation recognition dictionary and chooses Hownet sentiment dictionary. According to the research needs of this article, four vocabularies of positive evaluation words, positive emotion words, negative evaluation words, and negative emotion words in the Chinese dictionary are selected. Because the dictionary does not contain some Internet terms that frequently appear in common comments, such as V5, ding, liangle, and garbage. This article artificially adds these commonly used network emotion words when establishing positive sentiment evaluation vocabulary and negative sentiment evaluation vocabulary to improve the sentiment vocabulary.

###### Emotional Orientation Recognition Algorithm Based on Emotional Words

The sentiment orientation recognition algorithm based on sentiment words in this paper is a simple method of comparing the number of positive sentiment comments and negative sentiment comments contained in the review text, named SWM (Sentiment-word method). Since the algorithm is based on a dictionary, the first step in the algorithm is word segmentation, which uses the Jiyi Chinese word segmentation component. After word segmentation, the comment text is compared with the words in the positive sentimental vocabulary. It counts the number of words in the text that appear in the positive sentiment evaluation lexicon, denoted as S; after word segmentation, the comment text is compared with the words in the negative sentiment review lexicon, and the number of words in the text in the negative sentiment review lexicon is counted and recorded as T. Comparing the sizes of S and T, if S ≥ T, the text is determined to be a positive sentiment review text with a positive attitude; if it is determined to be a negative sentiment review text with a negative attitude.

##### Emotional Orientation Recognition Algorithm Based on Polynomial Bayesian Model

###### Overview of Bayes’ Theorem

In text classification, a certain text needs to be classified into a certain category, and several categories are often given during classification. Each text will have multiple characteristics, which can be described by the characteristics of the text.

It assumes that a certain text has n features, and each feature is represented by a vector. Then the text feature set can be represented by an n-dimensional vector $x=\left\{x_{1},x_{2},x_{3},\ldots ,x_{n}\right\}$ , where $x_{1},x_{2},x_{3},\ldots ,x_{n}$ is the n features in the text, and the set of *x* constitutes the training set feature set X. At the same time, it is assumed that there are m categories given in the classification, then category set $Y=\left\{y_{1},y_{2},y_{3},\ldots ,y_{m}\right\}$ , each text *x* can correspond to one or more categories in set Y.

Bayes’ theorem is based on probability statistics, and its main idea is based on samples and related data information. It calculates the posterior probability, and the attribution category of the test sample can be determined through the posterior probability.

###### Polynomial Bayesian Model

Bayes’ theorem is based on the assumption that text features are mutually exclusive, and this assumption is often not true in reality. If the text features are not independent of each other, it will lead to a high dimensionality of the text feature space and increase the time complexity and space complexity of the algorithm. According to the related knowledge of probability statistics, by observing the results of multiple experiments, it can be found that the probability of an event often obeys the normal distribution, Poisson distribution, binomial distribution, and other forms. Therefore, a polynomial model can be introduced on the basis of Bayesian theory to reduce the complexity of training.

##### Emotional Orientation Recognition Algorithm Based on Integrated Probabilistic Reasoning Model

###### Integrated Probabilistic Reasoning Model Classification Algorithm Framework

Online comments are the main form of emotional expression for the public to comment on news on the Internet and are an important data source for identifying the emotional tendencies of short online texts. Based on the short length of the short text, it contains less information. The classification algorithm framework of the integrated probabilistic reasoning model presented in this article contains the following parts:

1. It uses N-gram to extract the features in the training set and normalize it;
2. It uses principal component analysis to reduce the dimensionality of the feature space of the training set;
3. It uses the Ens-PRM method to build an ensemble classifier model;
4. It analyzes the classification results of each sub-classifier and makes the final classification judgment.

###### Probabilistic Reasoning Model

Probabilistic Reasoning Model (PRM) is a combination of PCA and Bayesian algorithm. PRM first uses PCA to compress the feature space to extract more discriminating features, and then evaluates the intra-class density under the random division of the feature space. The algorithm runs fast and is easy to implement.

The Bayesian classifier is one of the classic classifiers, which includes the conditional probability density evaluation function for each category, and requires a larger number of training sets to achieve better results. Use the likelihood rate to test the decision rules to obtain the minimum probability of error, is referred to as Bayesian error rate, bayes error rate is used to measure the discriminative capability of the features. The PRM algorithm uses the maximum posterior probability (MAP) as the classification criterion, and the optimal classification of the MAP decision rule is based on the minimum Bayesian error rate.

###### Integrated Probabilistic Reasoning Model

The integrated probabilistic reasoning model includes two parts: random feature subspace division and base classifier integration.

1. Feature subspace division
  In order to reduce the redundancy of the feature space and make full use of the discriminative information in the feature space, the sentiment orientation recognition algorithm based on the integrated probabilistic reasoning model uses a random subspace division method to divide the original feature space. This makes the subspace a series of N-Gram sequences containing emotion identification information. Ens-PRM uses a dynamic random partition method to divide subspaces, and then integrates corresponding PRM-based classifiers. The heuristic integrated classifier has a higher classification accuracy rate.
  Using the ensemble method, different base classifiers have complementary capabilities with each other. The classification error of a base classifier can be compensated by the classification result of another base classifier that is correctly classified, and the final ensemble classifier can obtain higher robustness than a single classifier. The random feature space division method is used to divide the feature space into several subspaces and form the corresponding base classifier. Among them, the number of base classifiers in the ensemble classifier and the dimension of each random subspace feature must be preset. For simplicity, we assume that the number of features in each subspace is the same, set the sampling rate to α, and the total number of features as Nf. Then the feature number Nfs in each feature subspace is floor (α*Nf), where floor is rounded down.
  This article assumes that the feature space division is based on the division of feature subsets, and the weight distribution of sample features DD is based on the uniform distribution of Nf. It is assumed that M times are randomly divided, and M feature subsets are obtained.
2. Ens-PRM algorithm
  Ens-PRM algorithm and integrated probabilistic inference model algorithm, the algorithm is described as follows:
  Enter:
  Sample $TS=\left\{\left(x_{1},y_{1}\right),\ldots ,\left(x_{N},y_{N}\right)\right\}$ of n labeled categories, where $y_{i}\in Y$ , category label $x_{i}\in X=\left\{1,\ldots ,N\right\}$ , *N* is the total number of categories, and *Y* is the sample space;
  And the number of base classifiers: M.
  Output:
  Integrated separator: H.
  Ens-PRM algorithm steps are as follows:

- Its initialized feature weight distribution DDi;
- According to the feature space division method mentioned above, the sample space is divided based on feature subsets, and the data set is reduced to $\tilde{S}=\left(TS,r\right)$ , where *r* is the dimension of the feature subspace;
- It returns $\tilde{S}$ as input to the learning algorithm mentioned above, trains the feature subspace, and outputs the base classifier hi;
- It repeats steps (b–c) M times to construct an integrated classifier $H=\left\{h_{1},\ldots ,h_{M}\right\}$ .

In short, the method of randomly dividing feature subspaces can set the number of divided spaces. For example, 30 and 50 are divided into 30 or 50 expert subsystems. The number of features in each subsystem is evenly distributed, and each subsystem recognizes the text to be tested. It calculates the average probability *p*1 for all subsystems to judge a certain text as a positive sample, and the average probability *p*2 for judgment as a negative sample. If *p*1 > *p*2, the text is a positive sample; if *p*1 < *p*2, the text is a negative sample.

It uses Bayesian formula to get the most possible target value:


(1) $$\begin{array}{c} v_{MAP}=\underset{v_{j}\in V}{\mathrm{argmax}}P\left(v_{j}|a_{1},a_{2},...,a_{n}\right)\\ =\underset{v_{j}\in V}{\mathrm{argmax}}\frac{P\left(a_{1},a_{2},\cdots ,a_{n}|v_{j}\right)P\left(v_{j}\right)}{P\left(a_{1},a_{2},\cdots ,a_{n}\right)}\\ =\underset{v_{j}\in V}{\mathrm{argmax}}P\left(a_{1},a_{2},\cdots ,a_{n}|v_{j}\right)P\left(v_{j}\right) \end{array}$$

The observed joint probability is equal to the product of the probabilities of a single attribute:


(2) $$P\left(a_{1},a_{2},\cdots ,a_{n}|v_{j}\right)=\underset{i}{\prod }P\left(a_{i}|v_{j}\right)$$

Then the target value of the naive Bayes algorithm is expressed as follows:


(3) $$v_{NB}=\underset{v_{j}\in V}{\mathrm{argmax}}P\left(v_{j}\right)\underset{i}{\prod }P\left(a_{i}|v_{j}\right)$$

The information gain Gain(T,E) of a relative sample set T of an attribute E is defined as:


(4) $$Gain\left(T,E\right)=Entropy\left(T\right)-\underset{v\in Value\left(E\right)}{\sum }\frac{T_{v}}{T}Entropy\left(T_{v}\right)$$

If the target attribute has a different values, then the entropy Entropy(T) of T relative to the category of a state is defined as follows, where wr is the proportion of *r* in T that belongs to the category:


(5) $$E\mathrm{ntropy}\left(T\right)=-\overset{a}{\underset{r=1}{\sum }}w_{r}\log_{2}w_{r}$$

When an output neuron *k* is iterating at *t*, its output error signal is defined as follows:


(6) $$e_{k}\left(t\right)=d_{k}\left(t\right)-y_{k}\left(t\right)$$

The instantaneous value of the entire error energy:


(7) $$\sigma \left(t\right)=\frac{1}{2}\underset{k\in T_{1}}{\sum }e_{k}^{2}\left(t\right)$$

Mean square error energy:


(8) $$\sigma_{AV}=\frac{1}{T_{0}}\overset{T_{0}}{\underset{t=1}{\sum }}\sigma \left(t\right)$$

The instantaneous value of the cost function degenerates to:


(9) $$\sigma \left(t\right)=\frac{1}{2}e^{2}\left(t\right)$$

In:


(10) $$e\left(t\right)=d\left(t\right)-x^{T}\left(t\right)\omega \left(t\right)$$

The formula of the simple linear classifier algorithm:


(11) ϖ(t+1)=ϖ(t)+αx(t)e(t)

The hypothesis to be learned is a function of the following form:


(12) $$\phi \left(x\right)=\omega_{0}+\overset{k}{\underset{u=1}{\sum }}\omega_{u}K_{u}\left(d\left(x_{u},x\right)\right)$$

Bell-shaped Gaussian kernel function:


(13) $$K_{u}\left(d\left(x_{u},x\right)\right)=e^{-\frac{1}{2\sigma_{u}^{2}}d^{2}\left(x_{u},x\right)}$$

The linear discriminant function is:


(14) λ(x)=εx+z

The classification is required to correctly classify all samples in the face of:


(15) $$y_{i}\left[\varepsilon x_{i}+b\right]-1\geq 0,i=1,2,\cdots ,n$$

The problem of seeking the optimal classification surface is transformed into a constrained optimization problem:


(16) $$\begin{array}{c} \min\;ise\frac{1}{2}w^{2}\\ subject\;to\;y_{i}\left[\varepsilon x_{i}+b\right]-1\geq 0\;\;\;i=1,2,\cdots ,l \end{array}$$

It uses the Lagrangian multiplier method:


(17) $$\beta_{i}\left\{y_{i}\left[\varepsilon x_{i}+b\right]-1\right\}=0$$

The optimal classification function solution of the problem is:


(18) $$f\left(x\right)=\mathrm{sgn}\left\{\varepsilon^{*}\cdot x+b^{*}\right\}=\mathrm{sgn}\left\{\overset{k}{\underset{i=1}{\sum }}\beta_{i}^{*}y_{i}\left(x_{i}\cdot x\right)+b^{*}\right\}$$

Adding a relaxation term $\delta_{i}\geq 0$ to condition $y_{i}\left[\varepsilon x_{i}+b\right]-1\geq 0$ , becomes:


(19) $$y_{i}\left[\varepsilon x_{i}+b\right]-1+\delta_{i}\geq 0$$

The corresponding classification function also becomes:


(20) $$f\left(x\right)=\mathrm{sgn}\left\{\overset{k}{\underset{i=1}{\sum }}\beta_{i}^{*}y_{i}K\left(x_{i}\cdot x\right)+b^{*}\right\}$$

## News Sentiment Communication

This article crawled relevant news comment texts from Sina News and Sohu News and collected a total of 200 news comment sets (including 108 Sina News and 92 Sohu News). As a result, the sentiment recognition process was applied to the set of comments in this paper, where duplicate comments and sentiment-irrelevant comments have been filtered out in the recognition process. The relevant statistical information of the data set used in the news sentiment communication experiment is shown in Table 1.

**Table 1:** Statistical information related to the dataset used in the news emotion communication experiment.

Data set name	Number of news articles	Total number of comments (positive/negative)	Average number of comment words per news article	Average number of sentiment words per review
Data_A_	348	1,561 (1,117/444)	56.05	9.68
Data_C_	36	975 (708/267)	59.29	10.39
Data_L_	334	616 (436/180)	51.02	8.60

**Figure 7:**
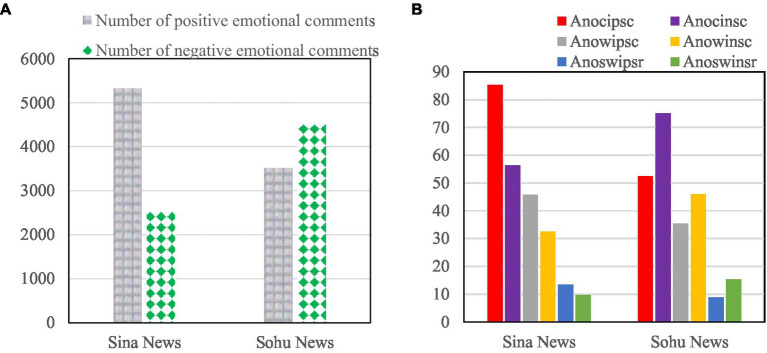
Information on the experimental dataset of news sentiment commentary. **(A)** Shows the number of positive/negative sentiment comments on Sina News and Sohu News. **(B)** Shows the average number of positive/negative.

The specific information of the final comment set is shown in Figure 7.

Figure 7A shows the number of positive/negative sentiment comments on Sina News and Sohu News. Figure 7B shows the average number of positive/negative sentiment characters (Anocipsc/Anocinsc), the average number of positive/negative sentiment words (Anowipsc/Anowinsc), and the average number of positive/negative sentiment words (Anoswipsr/Anoswinsr) of Sina News and Sohu News.

It constructs an emotional word vocabulary, as shown in Table 2.

**Table 2:** Distribution of words in the emotion word list among the four emotion resources.

Emotional vocabulary sources		Positive emotional words	Negative emotional words	Total words
Repetition for dictionaries (words from multiple dictionaries)	Student dictionary of positive and negative meanings	724	775	1,499
	HowNet	3,920	3,629	7,549
	NTUSD	2,537	7,240	9,777
	Sentiment.dict.v1.0	5,459	4,418	9,877
	Newly added vocabulary	104	287	391
No duplication of dictionaries (entries exist only from one dictionary)	Student dictionary of Positive and Negative Meanings	61	86	147
	HowNet	2,780	2,760	5,540
	NTUSD	1,648	6,089	7,737
	Sentiment.dict.v1.0	3,522	2,323	5,845
	Newly added vocabulary	104	287	391

When the unmarked rate is 0.5, the comparison results of the recognition algorithms are shown in Tables 3 and 4.

**Table 3:** Comparison results of recognition algorithms when the label-free rate is taken as 0.5 (base classifier is MNB).

Algorithm	K	PP (%)	RP (%)	FP (%)
AMVS	9.32	91.40 ± 0.2	92.85 ± 0.5	92.12 ± 0.4
PIVL	3	86.22 ± 0.8	92.87 ± 0.8	89.42 ± 0.8
KSE-cotraining	3	86.32 ± 0.3	92.57 ± 0.4	89.34 ± 0.4
Static cotraining(*K* = 10)	10	84.91 ± 0.6	91.33 ± 0.7	88.00 ± 0.7
Static cotraining(*K* = 20)	20	84.74 ± 1.1	86.34 ± 1.0	85.53 ± 1.1
Dynamic cotraining(*K* = 10)	10	88.28 ± 0.4	91.53 ± 0.4	89.88 ± 0.2
Dynamic cotraining(*K* = 20)	20	88.61 ± 0.7	90.31 ± 0.4	89.45 ± 0.6
Self-training	1	83.49 ± 0.5	88.64 ± 2.2	85.99 ± 1.2

**Table 4:** Comparison results of recognition algorithms when the label-free rate is taken as 0.5 (base classifier is SVM).

Algorithm	K	PP (%)	RP (%)	FP (%)
AMVS	7.27	92.10 ± 0.6	93.16 ± 0.4	92.63 ± 0.5
PIVL	3	85.69 ± 0.9	93.67 ± 0.6	89.50 ± 0.8
KSE-cotraining	3	85.54 ± 0.5	93.49 ± 0.9	89.34 ± 0.7
Static cotraining(*K* = 10)	10	88.47 ± 0.5	91.40 ± 0.8	89.91 ± 0.7
Static cotraining(*K* = 20)	20	89.93 ± 0.4	88.45 ± 0.6	89.18 ± 0.5
Dynamic cotraining(*K* = 10)	10	92.00 ± 0.5	88.02 ± 0.9	89.96 ± 0.4
Dynamic cotraining(*K* = 20)	20	91.10 ± 0.6	89.51 ± 1.1	90.30 ± 0.9
Self-training	1	86.51 ± 1.2	88.07 ± 1.8	87.28 ± 1.5

Tables 3 and 4 record the recognition results of all algorithms. Compared with other recognition algorithms, the algorithm in this paper shows higher accuracy.

**Figure 8:**
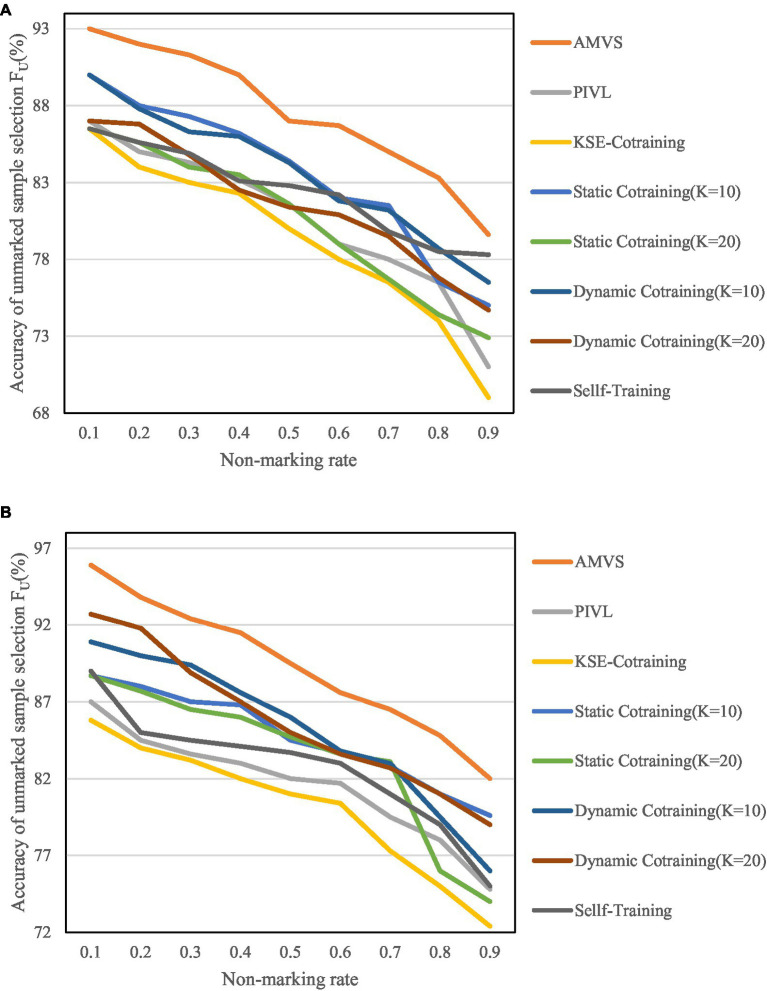
Variation of unmarked sample selection accuracy F_U_ at different unmarked rates. **(A)** Variation of F_U_ when the base classifier is MNB. **(B)** Variation of F_U_ when the base classifier is SVM.

**Figure 9:**
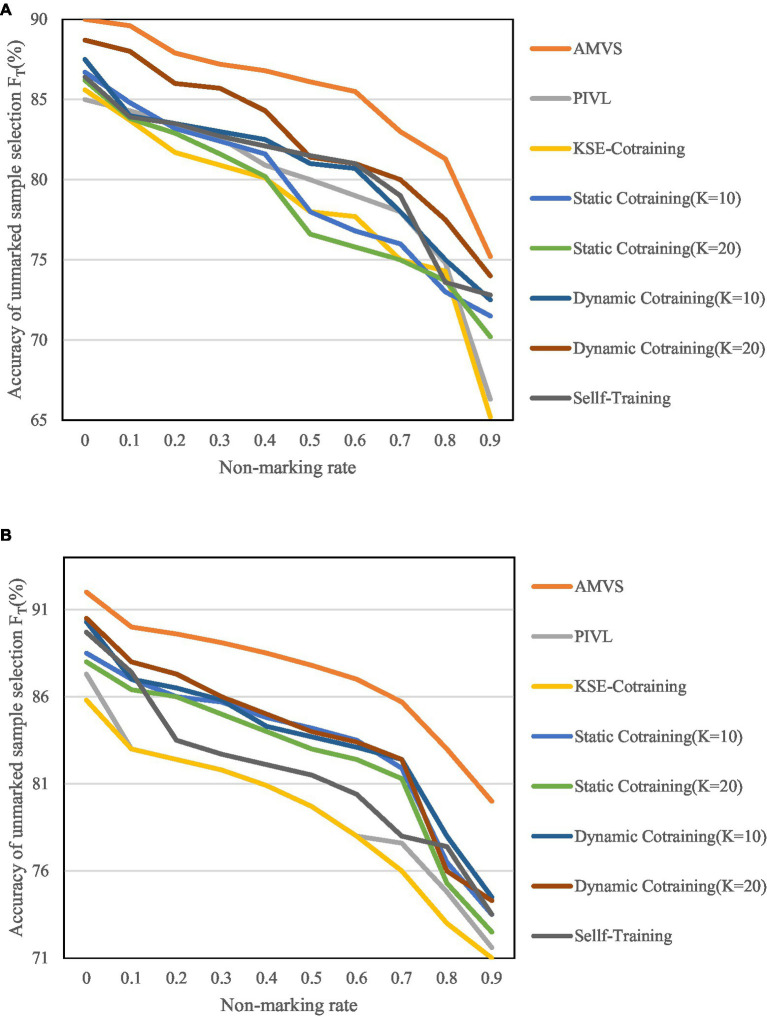
Variation of unmarked sample selection accuracy F_T_ at different unmarked rates. **(A)** Variation of F_T_ when the base classifier is MNB. **(B)** Variation of F_T_ when the base classifier is SVM.

According to the parameter settings when the best effect is achieved, the number of base classifiers K in the algorithm are, respectively, selected as 10 and 20 for experiment. The results are shown in Figures 8, 9.

Figures 8, 9 show that as the unlabeled rate continues to increase, the algorithm has shown high scalability and robustness in the screening of unlabeled samples, even when the unlabeled rate reaches 0.9. That is, when there are very few labeled samples in the training set, the above recognition accuracy can also be achieved.

The parameter settings of different recognition algorithms are shown in Table 5.

**Table 5:** Parameter settings of different recognition algorithms.

Algorithm	Feature view sampling rate	Sampling guidelines	K	*ρ*
AMVS	Variable	W, D distribution	Variable	0.6
PIVL	N/A	Subjective/objective sentence division	3	0.6
Dynamic cotraining	0.5	Uniform distribution	4/10/20	0.6
Static cotraining	0.5	Uniform distribution	3/10/20	0.6
KSE-cotraining	N/A	Emotion/detail sentence division	3	0.6
Self-training	N/A	N/A	1	0.6

**Figure 10:**
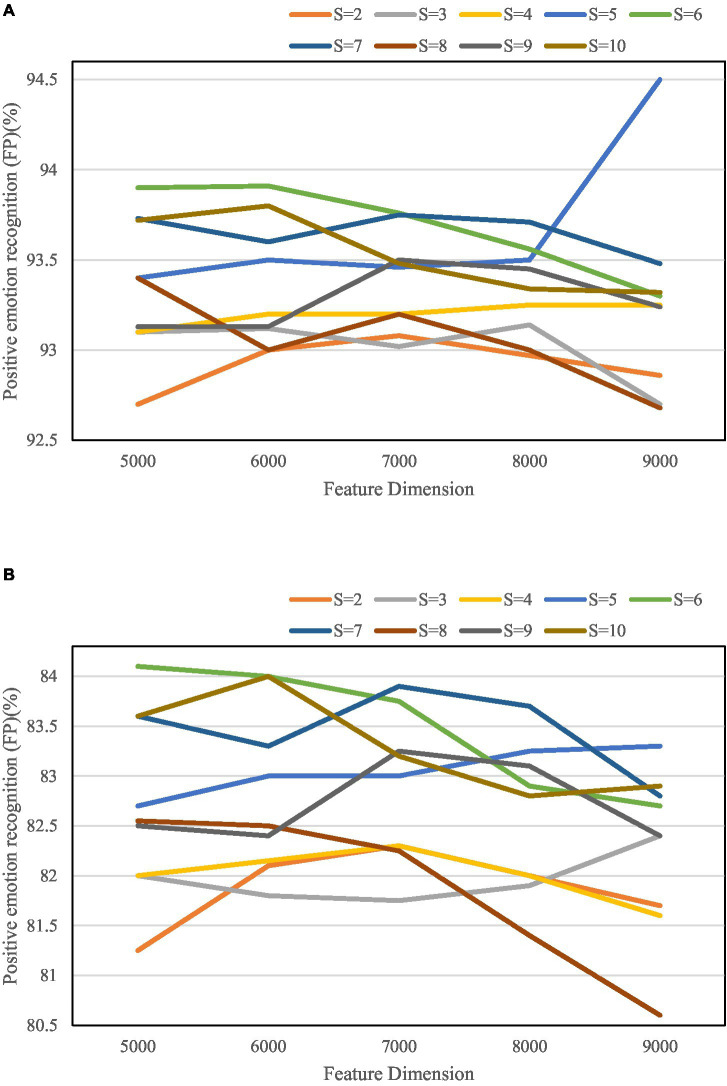
Micro-averaged *F*-value results for positive/negative sentiment sample identification. **(A)** Micro-averaged *F*-value results for positive sentiment sample identification. **(B)** Micro-averaged *F*-value results for negative sentiment sample identification.

It tests the effect of the number S of cross-divided check subsets on the performance of the algorithm, and the specific results are shown in Figure 10.

It can be seen from Figure 10 that in all the parameter combination conditions, the algorithm can not only reduce the feature dimensionality but also ensure the accuracy of recognition with a probability of 89%.

## Discussion

In order to solve the problem of manual labeling of training examples, this article provides a polygon-based adjustable emotion recognition algorithm. The algorithm introduces emotional vocabulary to calculate the emotional weight of N-gram attributes. It selects the corresponding attributes according to the emotional weight distribution data and adjusts the saliency distribution of the attributes in the repeated selection process. For each visual sample, after completing the training process multiple times during the inspection process, the sample with the highest label reliability is selected to refine the training. It is demonstrated that the angle by which the features are selected by the algorithm in this paper is more versatile by comparison with many traditional algorithms. A more reliable and accurate labeling of its unlabeled samples.

## Conclusion

This paper designs and implements an emotional orientation recognition algorithm (Ens-PRM) based on an integrated probabilistic inference model. In the experimental comparison between Ens-PRM and a simple dictionary-based algorithm, we have demonstrated that the recognition accuracy of the dictionary-based algorithm is not high, but also it is susceptible to the interference of objective emotions and has poor generality. It can be seen from the experimental results that Ens-PRM has achieved better classification results. But Ens-PRM is more time-consuming, when the data set is huge, Ens-PRM takes longer. Therefore, research is needed to improve the operation speed of Ens-PRM in the future.

## Data Availability Statement

The original contributions presented in the study are included in the article/supplementary material, further inquiries can be directed to the corresponding author.

## Author Contributions

The author confirms being the sole contributor of this work and has approved it for publication.

## Conflict of Interest

The author declares that the research was conducted in the absence of any commercial or financial relationships that could be construed as a potential conflict of interest.

## Publisher’s Note

All claims expressed in this article are solely those of the authors and do not necessarily represent those of their affiliated organizations, or those of the publisher, the editors and the reviewers. Any product that may be evaluated in this article, or claim that may be made by its manufacturer, is not guaranteed or endorsed by the publisher.
